# Determination of ****β****-Galactooligosaccharides by Liquid Chromatography

**DOI:** 10.1155/2014/768406

**Published:** 2014-02-26

**Authors:** Sean Austin, Thierry Bénet, Julien Michaud, Denis Cuany, Philippe Rohfritsch

**Affiliations:** ^1^Nestlé Research Centre, 1000 Lausanne, Switzerland; ^2^Novartis Consumer Health S.A., 1260 Nyon, Switzerland; ^3^Nestlé Waters Supply Est-Vosges, Contrex Factory, 306 rue de Lorraine, 88140 Contrexeville, France

## Abstract

Beta-galactooligosaccharides (GOS) are oligosaccharides normally produced industrially by transgalactosylation of lactose. They are also present naturally in the milk of many animals including humans and cows. GOS are thought to be good for health, being potential prebiotic fibres, and are increasingly added to food products. In order to control the GOS content of products, the AOAC official method 2001.02 was developed. However, the method has some shortcomings and in particular is unsuited to the analysis of products containing high levels of lactose such as infant formula. To overcome this problem, we developed a new method for application to infant formula and tested it on various GOS ingredients as well as infant formulae. When applied to GOS ingredients the results of the new method compare well with those of the official AOAC method, typically giving results in the range 90–110% of those of the official method and having an expanded measurement uncertainty of less than 15%. For three products, the results were outside this range (recoveries of 80–120% and expended measurement uncertainties up to 20%). When applied to the analysis of infant formula, recoveries were in the range of 92–102% and the expanded measurement uncertainties were between 4.2 and 11%.

## 1. Introduction

Beta-galactooligosaccharides (GOS) are oligosaccharides composed primarily of galactose and often terminate with a glucose residue at the reducing end. They occur naturally in the milk of many animals [1] including humans [2], cows [3], and wallabies [4]. Industrial production of GOS can be achieved in several ways, but most commercial products are produced from lactose using *β*-galactosidase under conditions which favour transgalactosylation [5–7].

The structures of GOS produced by enzymes of different bacteria have been studied [8, 9] as have those from commercial GOS production [10, 11]. In all cases, the GOS are predominantly composed of a chain of *β*-D-galactose residues terminating at the reducing end with a D-glucose residue. The chains typically contain between 2 and 8 monosaccharide residues. They are predominantly linear, but a few branched structures have been reported [9]. In some instances, the reducing end was galactose, and a few terminated with fructose. Coulier et al. [10] studied one commercial GOS (Vivinal GOS) and demonstrated the predominance of the (1 → 4)-linked *β*-D-Gal residue in the oligosaccharides but other linkages such as (1 → 6)-linked *β*-D-Gal and (1 → 3)-linked *β*-D-Gal were also observed. On the shorter oligosaccharides (di- and trisaccharides), the reducing end glucose could be linked through position 2, 3, 4, or 6 but as the oligosaccharide chain length increased, linkage through the 4 position predominated. Coulier et al. [10] also reported the presence of 2 nonreducing disaccharides, *β*-D-Gal-(1↔1)-*β*-D-Glc and *β*-D-Gal-(1↔1)-*α*-D-Glc.

Hernández-Hernández et al. [11] studied the glycosidic linkage types present in three commercial GOS samples (Vivinal, Bimuno, and Yum Yum). They determined the linkages only via MS fragmentation data; therefore the anomeric configuration could not be confirmed. The relative abundances of the different linkage types are difficult to discern from their data due to coelution of different oligosaccharides in their LC system. Nevertheless, all three GOS contained (1 → 6)-linked, (1 → 3)-linked, and (1 → 4)-linked *β*-D-Gal residues in varying abundance. The (1 → 2)-linked *β*-D-Gal residue was less common but was present in all three samples.

GOS are not hydrolysed in the upper gastrointestinal tract of humans, but they enter the large intestine and can be metabolized by the colonic microflora [12]. A number of studies have indicated that GOS consumption may alter the microflora population by selectively increasing the number of certain types of bacteria, in particular bifidobacteria [13–19], but this has not been the case in every study [20]. Should it be accepted that this modulation of the gut microflora induces a health benefit, these oligosaccharides may be considered as prebiotics. It has also been reported that GOS may reduce adhesion of pathogenic bacteria or their toxins to the host receptors, potentially protecting the host against illness via other mechanisms [21–23]. If it is considered that there is sufficient evidence that GOS provide some health benefit, then, in certain markets, they may also be considered as dietary fibres. In 2009, after more than 16 years of discussions, CODEX settled on a new definition of dietary fibre [24]. Unfortunately a single definition was not proposed, instead two were proposed. The difference between the two definitions was the chain length of the carbohydrate polymer that could be considered as fibre. In one, the carbohydrate polymer must consist of 10 or more monomeric units; in the other, the carbohydrate polymer must consist of three or more monomeric units. Individual countries remain free to choose which definition they want to apply. GOS ingredients are generally mixtures of oligosaccharides having chain lengths between two and eight monomeric units [25]. GOS therefore cannot be considered as fibre in countries using the minimum chain length of 10 monomeric units. On the other hand, in markets that adopted the definition of three monomeric units or more (such as Australia, New Zealand, and the European Union), they may be considered fibres. However, the GOS contain nondigestible disaccharides that, while potentially providing a prebiotic effect or other health benefit [12, 26–28], do not fall within the definition of dietary fibre. It would therefore be necessary to exclude these GOS disaccharides when declaring GOS as a fibre for product labeling.

Few methods for quantitative analysis of GOS have been reported in literature. The current AOAC 2001.02 method [29] for GOS analysis is based on the work of de Slegte [25] and is the only fully validated official method for determination of GOS in food samples. It uses a hydrolysis to convert the GOS to galactose and glucose, and then measurements of the released monosaccharides are used to calculate the GOS content. This method has two limitations: (1) using this method, it is not possible to distinguish the GOS that may fall under the definition of fibre from those that do not and, (2) in products containing high levels of free galactose or lactose, and containing relatively low concentrations of GOS, a small error made on the free galactose or lactose measurement will induce a large error on the GOS measurement (as would be the case in infant formula e.g.).

Coulier et al. [10] and Hernández-Hernández et al. [11] produced some quantitative data to estimate the relative amounts of oligosaccharides of each DP in the GOS products they studied. However quantitation was not the primary aim of their work, hence there was no validation of their methodology and it was not applied to complex food matrices. Albrecht et al. [30] developed a method for GOS analysis using capillary electrophoresis with laser-induced fluorescence (CE-LIF). The method developed would overcome many of the obstacles with the AOAC method. Unfortunately, they performed limited validation, and CE-LIF is not a common technology in food analysis laboratories. In this paper, we describe a method for GOS analysis that overcomes the two limitations of the AOAC 2001.02 method [29] and uses more commonly available instrumentation (HPLC) than CE-LIF.

## 2. Experimental

All chemicals were purchased from Sigma (Buchs, Switzerland) or Merck (Darmstadt, Germany) unless otherwise specified. All the water used was 18 M′*Ω* deionised water produced by a Milli-Q system (Millipore, Billerica, MA, USA). All of the oligosaccharides were purchased from Dextra Laboratories (Reading, UK) with the exception of lactose, maltotriose, and laminaritriose (Sigma). Galactooligosaccharide ingredients were obtained from FrieslandCampina (Amersfoort, The Netherlands), Yakult Pharmaceutical Industry (Tokyo, Japan), Kerry Group (Tralee, Ireland), Clasado (Sliema, Malta), Ingredion Inc. (Westchester Illinois), and Promovita Ingredients Ltd (Crewe, UK). The GOS samples have been coded GOS-1A, GOS-1B, GOS-2, GOS-3A, GOS-3B, GOS-4, GOS-5, and GOS-6 (in no specific order). GOS-1A and GOS-1B are two different formats (e.g., powder and syrup) of product from the same supplier. GOS-3A and GOS-3B are also different formats of product from the same supplier.

### 2.1. Determination of GOS by Reference Method

The GOS contents of GOS ingredients were determined using the AOAC official method AOAC 2001.02 [29].

### 2.2. Determination of GOS by HPAEC-PAD Profiling Method

For determination of GOS in infant formula, a sample of the GOS ingredient used in production was first analysed by the AOAC method [29]. A solution of the ingredient (0.6 g/100 mL) was profiled by HPAEC-PAD on an ICS 3000 system (Dionex, Olten, Switzerland) equipped with a CarboPac PA 100 column (250 × 4 mm, Dionex). An aliquot (25 *μ*L) of the solution was injected onto the column and eluted at 1 mL/min with a linear gradient of sodium acetate (10–100 mmol/L in 30 min) in sodium hydroxide (90 mmol/L) and marker peaks were selected at around 9 and 13 min. A solution of infant formula (20 g/L) was prepared and an aliquot (25 *μ*L) was injected on the same HPAEC-PAD system using the same method. By comparing the areas of the two marker peaks in the infant formula sample and in the GOS ingredient, it was possible to determine the GOS content of the infant formula.

### 2.3. Determination of Total Oligosaccharides by HPLC after Labeling with 2-Aminobenzamide

Samples of GOS ingredient (250 mg) were dissolved to 100 mL in water. Samples of infant formula (2 g) were dissolved to 50 mL in water. A 500 *μ*L aliquot of sample solution was taken and 200 *μ*L of laminaritriose solution (0.3 mmol/L) was added. The mixture was mixed using a vortex mixer, and then a 20 *μ*L aliquot was transferred to a 2.0 mL microcentrifuge tube and labelled with 2-aminobenzamide (2-AB) following the protocol of Bigge et al. [31] with some modifications previously described by Bénet and Austin [32]. Briefly, 200 *μ*L of 2-AB reagent (0.35 mol/L 2-AB and 1.0 mol/L sodium cyanoborohydride in dimethylsulfoxide containing 30% acetic acid) was added to the aliquot and mixed well. The solution was heated at 60°C for 2 h and then cooled on ice. 1.5 mL of acetonitrile/water (75/25) was added and the solution was transferred to a vial suitable for the HPLC autosampler.

If samples contained (or were suspected to contain) maltodextrins, then 1.0 mL of ammonium acetate buffer (0.1 mol/L pH 5.5) was added after the labeling reaction (but before the addition of acetonitrile/water). An aliquot (0.5 mL) of the solution was transferred to a 2 mL tube and 200 *μ*L of amyloglucosidase (60 U/mL in 0.1 mol/L ammonium acetate, pH 5.5) was added. The solution was then incubated at 50°C for 30 min. Samples were then cooled to room temperature and diluted with 0.70 mL of acetonitrile.

Labelled oligosaccharides were cleaned and separated using an HPLC (Ultimate 3000 RS, Dionex, Sunnyville, CA, USA, or a Prominence, Shimadzu, Tokyo, Japan) in the configuration described previously [32] on TSK Gel Amide-80 guard (3.2 × 15 mm, 3 *μ*m) and analytical (4.6 × 150 mm, 3 *μ*m) columns (Tosoh Bioscience, Stuttgart, Germany). Detection was performed by a Dionex RF-2000 or Shimadzu RF-10Axl fluorescence detector using *λ*_ex_ = 330 nm and *λ*_em_ = 420 nm. Eluent A was 100% acetonitrile. Eluent B was 50 mmol/L ammonium formate, pH 4.4. A 10 *μ*L aliquot (or 20 *μ*L aliquot for amyloglucosidase treated samples) of the labelled OS solution was injected onto the guard cartridge under isocratic conditions (98% A) at a flow rate of 1 mL/min for 7.5 min. The eluent from the guard cartridge was then directed onto a TSK gel amide-80 analytical column (4.6 × 150 mm, 3 *μ*m, Tosoh Bioscience) held at 23°C and the mobile phase composition was ramped to 84% A over 0.5 min. Oligosaccharides were then separated under the following conditions: 84% A from 8 to 16 min, followed by a linear gradient to 61% A at 50 min. At 51 min, the flow rate was dropped to 0.8 mL/min and the eluent composition was held at 20% A for 3 min to wash the column. The composition was returned to 90% A over 1 min and then the flow rate was returned to 1.0 mL/min and the column reequilibrated under those conditions for 6 min before returning the system to the load conditions for the next sample.

To determine the molecular mass of the OS responsible for each chromatographic peak, the same procedure as above was followed, but the starting concentrations of the samples were 2-3 times greater, and the injection volume was varied between 5 and 100 *μ*L to achieve sufficient concentration for MS detection. The effluent from the analytical column was split approximately 60/40, and the flow at around 400 *μ*L/min was sent to the mass spectrometer, while the remaining flow went to the fluorescence detector using the parameters described above. The mass spectrometer was an API 4000 Q-TRAP (AB Sciex, Foster City, CA, USA) equipped with a turbo ion spray source controlled by Analyst 1.5 (AB Sciex). The HPLC was an Ultimate 3000 (Dionex) controlled by Analyst 1.5 (AB Sciex) via DCMS link (Dionex). MS source parameters were set as follows: curtain gas (CUR): 17.0, ion source temperature (TEM): 400°C, nebulizer gas (GS1): 60.0, Heater Gas (GS2): 20.0, Interface Heater (Ihe): On, Ion Spray Potential (IS): −3800 V, declustering potential (DP): −100, and entrance potential (EP): −10. The experiment was run in multiple ion monitoring (MIM) mode, using a list of the *m*/*z* ratios of the [M–H]^−^ ions covering disaccharides to nonasaccharides. For each mass, a dwell time of 50 to 70 ms was set.

To determine OS concentration, each peak in the fluorescence chromatogram was integrated and the peak area (relative to the internal standard) was compared to that of a calibration curve (produced using different concentrations of maltotriose with laminaritriose as internal standard). This resulted in a molar concentration for each component in the chromatogram. These were then converted to mass concentrations by conversion using the molecular weight (assigned from the MS experiments).

### 2.4. Method Validation

The methods were validated by assessing linearity of the calibration curve, the method accuracy, and method precision (repeatability and intermediate reproducibility).

*Linearity* was assessed in the HPAEC-PAD profiling method by injecting a series of different concentrations of GOS ingredient and plotting the area of the two markers against the GOS concentration.

*Linearity *was assessed for the HPLC-FLD method by plotting the ratio (standard/IS) of the peak areas against the ratio of concentrations (standard/IS) using different concentrations of maltotriose as the standard and a fixed concentration of laminaritriose (300 nmol/mL) as the internal standard.

*Repeatability *(*r*)* and intermediate reproducibility *(iR) were assessed by analysing samples (GOS or infant formula containing GOS) in duplicate on at least 6 different days. SD (*r*) and SD  (iR) were then calculated using the following formulae:

(1) ClassicalRobustSD(r)SD(r)=∑i=1nSDi2n=∑i=1n(xi1−xi2)22nSDrob(r)=1.0484×Medi=1,…,n{|xi1−xi2|}SD(iR)SD(iR)=SD2(b)+12×SD2(r)SDrob(iR)=SDrob2(b)+12×SDrob2(r),

where

 
*n* is the number of (single or duplicate) determinations,
 Med {*x*_*i*_} is the median of the specified set of results {*x*_*i*_},
 
*x*_*i*_ is the individual result within the set of single determinations with *i* going from 1 to *n*,
 
*x*_*i*1_ and *x*_*i*2_ are the two results within the set of duplicate determination with *i* going from 1 to *n*,
   SD_*i*_ is the standard deviation within the set of duplicates/replicates with *i* going from 1 to *n*,
 SD  (*b*) is the standard deviation between the means of duplicates, and SD_rob_  (*b*) is the robust standard deviation between the means of duplicate.

*Recovery *was determined for GOS samples by comparing the result of the new method against that obtained by the AOAC official method [29]. For infant formula samples, the recovery was assessed by spiking blank infant formula with GOS. The recovery was calculated by subtracting the GOS content measured in a blank formula from that measured in a spiked formula and dividing the result by the amount of GOS spiked into the sample. The result was then expressed as a percentage.

*Measurement Uncertainty *was calculated by combining the results from the recovery experiment with those from the precision experiment as described by Barwick and Ellison [33].

All statistical calculations were performed using the in-house statistical package QStat.net using both classical and robust statistics.

## 3. Results

**Figure 1:**
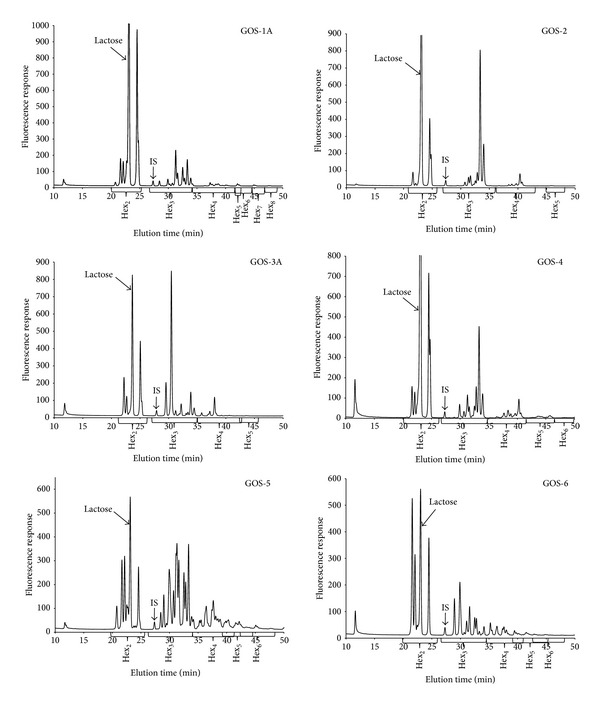
HPLC-FLD profiles of different GOS products.

Using the HPLC-FLD method, each type of GOS gave rise to a distinct GOS profile (Figure 1) except those from the same supplier which had identical profiles (data not shown). The data from LC-MS experiments were used to assign the mass (and hence chain length) for each peak in the chromatogram. Separation of the major oligosaccharides by chain length was achieved, but, in 3 samples (GOS-6, GOS-5, and GOS-1), coelution of some minor signals was observed; mostly some Hex_5_ were not completely resolved from some Hex_4_ or Hex_6_. The disaccharide area of the chromatograms is always well resolved from the areas containing longer chain GOS, thus enabling the separation of GOS matching one of the CODEX fibre definitions from the GOS that does not.

**Figure 2:**
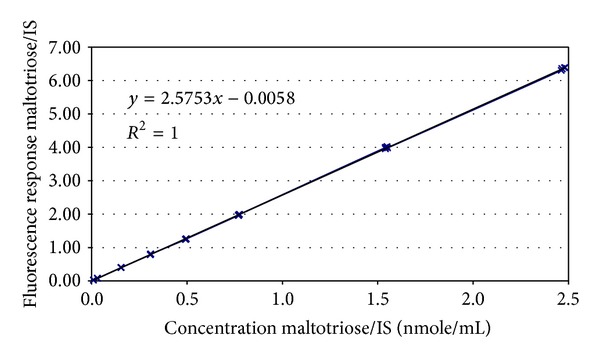
Calibration curve prepared using maltotriose.

Standards of each individual GOS are not available; therefore, quantitation cannot be performed in the usual way using standards for each individual analyte. However, since each chain has been labelled with 2-AB and it is the 2-AB which is detected by the fluorimeter, we can exploit this for quantitation. Bigge et al. [31] already demonstrated that labeling with 2-AB is quantitative for a broad range of different oligosaccharides; thus, it should be possible to perform molar quantitation based on a calibration curve produced using any suitable 2-AB labelled oligosaccharide. We selected maltotriose as our calibrant and used laminaritriose as an internal standard (IS). A standard curve was prepared by plotting the relative response of the calibrant to the IS against the relative concentration of the calibrant to the IS (Figure 2). The curve was found to be linear in the range from 3 to 750 nmol/mL when using an IS concentration of 300 nmol/mL and an injection volume of 10 *μ*L. The curve was then used to calculate the molar content of GOS in each area of the chromatogram; this was then converted to mass concentrations by multiplying by the molar mass of the peaks (as determined in the LC-MS experiment). In the few cases where there were peaks containing oligosaccharides of 2 different masses, the lower mass was assigned to the whole peak. The results obtained on GOS ingredients using the profiling method were compared against those obtained using the AOAC method 2001.02 (Table 1). In most cases, the new method produces results within the range 90–110% of the current AOAC method, but statistical analysis (*t*-test) indicates that, in most cases, the results of the two methods are different (with the exceptions of GOS-2 and GOS-6 for which the *t*-test indicates that the 2 methods give equivalent results). However, there are three samples for which the new method gives results outside the 90–110% window. The GOS content of the GOS-1A and B products seems to be overestimated (117–120%) using the new HPLC-FLD method, and the GOS content of the GOS-5 product seems to be underestimated (84%) using the HPLC-FLD method.

Since the AOAC 2001.02 method is not applicable to infant formula (lactose levels are too high), two approaches were developed for GOS analysis in such matrices. Initially, an HPAEC-PAD profiling method was developed. This method is simple, since it is a case of comparing the peak areas of 2 marker peaks in the GOS ingredient profile with the area of the same marker peaks in the formula. Recoveries were assessed using spiked infant formula and were in the region 94–99% (Table 2). Spiked formulae were also analysed using the HPLC-FLD method (Table 2); in this case, recoveries were in the range 92–102%.

The precision of the HPLC-FLD method applied to GOS ingredients was determined by analysing each ingredient in duplicate on six different days on the same instrument by the same operator. The data from these experiments were used to determine the relative standard deviation under repeatability conditions (RSD (*r*)) and the relative standard deviation under intermediate reproducibility conditions (RSD (iR)); results are shown in Table 3. The same data were also used to determine the total dietary fibre (TDF) content of the GOS ingredients by excluding the GOS disaccharides, and the precision of those measurements was also determined (Table 4). For GOS analysis, the robust RSD (*r*) is in the range 0.4–2.0% and the robust RSD(iR) is in the range 0.7–3.0%. For TDF analysis, the robust RSD (*r*) is in the range 0.3–2.3% and the robust RSD (iR) is in the range 1.1–3.0%.

The precision of the HPLC-FLD method applied to the analysis of GOS containing infant formulae was determined by analysing two commercially available formulae on eight different days in duplicate, on two different instruments using two different columns, and by two different operators (Table 5). The robust RSD(*r*) is in the range 0.4–0.8% and the robust RSD(iR) is in the range 1.1–2.0%. The HPAEC-PAD profiling method was performed on one formula on nine different days on a single instrument on the same column by one operator (Table 5). The robust RSD(*r*) was 2.4% and the robust RSD(iR) was 3.5%.

Measurement uncertainty (MU) was calculated according to the methods proposed by Barwick and Ellison [33] combining precision and recovery data (Table 6). The relative expanded MU for the analysis of GOS in infant formula was between 4 and 11% and for GOS in GOS ingredients was between 4.6 and 20%. The higher MUs were obtained for the products with poorer recoveries (Table 6), that is, GOS-1A, GOS-1B, and GOS-5. For the other GOS products, the relative expanded MU ranged from 4.6 to 13%.

## 4. Discussion

Each of the GOS products had a different oligosaccharide profile; the exceptions are the products from the same supplier but available in different formats (e.g., syrup or powder), that is, GOS-1A and GOS 1B and GOS-3A and GOS-3B. The different oligosaccharide profiles may (or may not) have some impact on the biological effects of the different types of GOS, but that remains to be determined. However, there is a significant impact for the TDF content of the different GOS. The disaccharide component of the GOS can represent between 15 and 50% of the total GOS depending on the product, meaning that the TDF fraction of the GOS varies between 50 and 85%. This is important information and has consequences for the labeling of GOS-containing products. Using the current AOAC 2001.02 method [29] for GOS analysis, 100% of the GOS would erroneously be considered as dietary fibre. However, using the HPLC-FLD method described here, it is possible to differentiate the GOS fraction having a DP ≥ 3 from the GOS disaccharides and thus the contribution of the GOS to TDF can be accurately assessed. In addition, the method enables the quantitation of the different groups of GOS according to the degree of polymerization which may be useful for quality control purposes or when trying to understand biological functions. In Table 7, the total GOS content has been normalized to 100% in order to compare the relative proportions of the oligosaccharides of different chain length and in addition contains the same data from some previous studies [10, 11, 30]. There is quite a lot of variation between the GOS from different suppliers, although the distributions of chain lengths from the same supplier are comparable.

**Figure 3:**
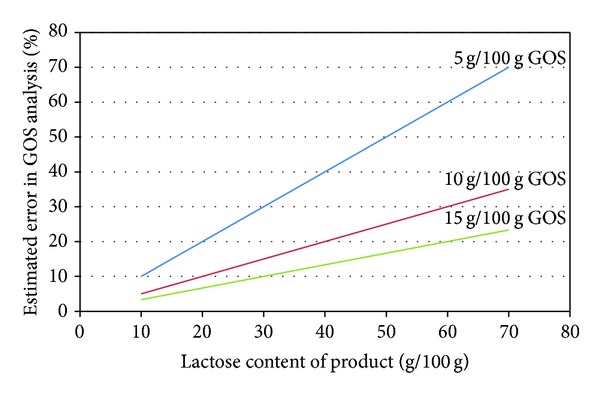
Estimated error on GOS determination by AOAC 2001.03 depending on lactose and GOS concentration assuming a 5% error on the lactose measurement.

The application of AOAC 2001.02 for the analysis of GOS in lactose-containing products is not a problem if the GOS content is high and/or if the lactose content is low.  Figure 3 shows the estimated error of the GOS analysis depending on the concentration of lactose and GOS assuming a 5% error in the determination of lactose. The graph demonstrates how rapidly the error in the GOS analysis increases, if the GOS content of a product is low. Infant formulae have a high lactose content, and their GOS contents are typically below 10 g/100 g; it is clear from Figure 3 that such products cannot be accurately analysed using the AOAC 2001.02 method [29]. We developed two methods to overcome this issue. The first method based on HPAEC-PAD profiling works well. However, it has the disadvantage that the analysing laboratory would need access to both the product for analysis as well as the appropriate GOS ingredient to perform the analysis. Furthermore, it requires the lab to perform four chromatographic runs for a single product: (1) the determination of free galactose and lactose in the ingredient, (2) the determination of total galactose in the ingredient after hydrolysis, (3) the profile of the GOS ingredient to determine the marker peak intensity versus concentration, and (4) the profile of the product to find the marker peaks to determine GOS concentration in the finished product. Such a process takes some time. The HPLC-FLD method has the advantage that only a single run is required and the analysing laboratory does not need access to the GOS ingredient. The disadvantages of the HPLC-FLD method are that the GOS must be derivatised before analysis and if the product contains other reducing oligosaccharides, these may interfere. GOS is often combined with inulin or FOS in infant formula. Fortunately, such oligosaccharides are either nonreducing or they have a fructose at the reducing end. The conditions used for labelling the oligosaccharides are such that ketoses (such as fructose) are not labelled, and thus fructans do not interfere with the analysis. Other oligosaccharides such as maltodextrins can be enzymatically hydrolysed to their monosaccharides to avoid that they interfere.

The performance of the HPLC-FLD method is quite good for most products (both formula and GOS ingredients) with a few exceptions. The major contributor to the measurement uncertainty tends to be the recovery because the recovery is significantly different from 100%. This is particularly a problem for GOS-1A, GOS-1B, and GOS-5. The method underestimates the GOS content for GOS-5 and also that of GOS-4. In fact, it is surprising that the method does not underestimate the GOS content in more cases. Knowing that the labelling reaction does not work on nonreducing oligosaccharides or on oligosaccharides containing a ketose at the reducing end, underestimation would be expected since detailed GOS analyses [10, 11] have revealed the presence of both nonreducing GOS and GOS that terminate in a fructose. The disaccharide region of the chromatogram around lactose is also quite busy, and, in some cases, there may be GOS disaccharides coeluting with the lactose that have not been determined. Apparent overestimation of the GOS content, as seems to be the case for GOS-1A, GOS-1B, GOS-3A, and GOS-3B, is more difficult to understand. A possible explanation may be that the reference value (obtained by AOAC 2001.02 [29]) has actually underestimated the GOS content. The AOAC method requires that all GOS disaccharides are well resolved from lactose when performing the free sugars part of the analysis. Some products may contain GOS disaccharides that coelute with the lactose in the reference method, leading to an overestimation of lactose and consequently an underestimation of GOS. Such a situation may not have been encountered during the development of AOAC 2001.02 depending on the type of GOS product used for the development.

## 5. Conclusion

This work was done to address the two major issues with the current AOAC 2001.02 method for GOS determination, that is, the difficulty in applying it to products containing large amounts of lactose and the incompatibility of the method with the current definition of dietary fibre. The HPLC-FLD method described here overcomes both of these issues and the expanded measurement uncertainty of the method is below 15% in most cases. Nevertheless, there appear to be a few GOS products for which the new method is not optimized. The precise cause of these problems needs further investigations to resolve.

## Figures and Tables

**Table 1:** Comparison of GOS content measured in different products using the AOAC method and the new method and comparison of results using both classical and robust statistics.

Sample	Reference value		Results (g/100 g)				Results (g/100 g)			
	(g/100 g)		Robust				Classical			
	Mean	SD	Median	SD	Recovery (%)	*P*	Mean	SD	Recovery (%)	*P*
GOS-1A	37.32	0.56	43.51	1.15	117*	0.001	43.54	1.57	117*	0.001
GOS-1B	36.26	1.32	43.75	1.81	121*	0.006	43.52	1.01	120*	0.007
GOS-2	39.53	0.40	39.42	1.17	99.7	0.861	39.66	1.08	100	0.839
GOS-3A	38.46	0.20	42.38	1.03	110*	0.000	42.27	0.666	110*	0.000
GOS-3B	51.45	0.34	54.11	1.04	105*	0.005	54.13	0.799	105*	0.003
GOS-4	28.70	1.06	26.00	0.274	90.6*	0.038	26.03	0.205	90.7*	0.040
GOS-5	79.02	1.11	66.06	0.382	83.6*	0.000	66.56	1.69	84.2*	0.000
GOS-6	44.66	0.76	43.12	0.555	96.6	0.101	43.06	0.447	96.4	0.088

*Indicates recoveries which are significantly different from 100% (*P* < 0.05).

**Table 2:** GOS recovery measured in spiked infant formulae.

Formula	Method	Nonspiked OS concentration (g/100 g)	Spiking level 1 (2.6 g/100 g) Recovery (%)	Spiking level 2 (5.1 g/100 g) Recovery (%)	Spiking level 3 (10.3 g/100 g) Recovery (%)
Infant formula	HPLC-FLD	0.18	100.8–102.0	97.3–97.6	92.1–96.7
H.A. formula	HPLC-FLD	0.37	92.2–94.9	95.1–95.8	92.7–95.7
Infant formula	HPAEC-PAD	n/a	94.6–99.3	n/a	n/a
H.A. formula	HPEAC-PAD	n/a	n/a	n/a	n/a

H.A. formulae are hypoallergenic formulae containing partially hydrolyzed proteins. n/a: not analysed.

**Table 3:** Precision of GOS analyses in ingredients using both robust and classical statistics.

Sample	GOS concentration (g/100 g)		RSD (*r*) (%)		RSD (iR) (%)	
	Median	Mean	Robust	Classical	Robust	Classical
GOS-1A	43.5	43.5	0.7	1.0	2.7	3.7
GOS-1B	43.7	43.5	0.4	0.9	2.7	2.4
GOS-2	39.4	39.7	0.9	1.0	3.0	2.8
GOS-3A	42.4	42.3	1.1	0.9	2.5	1.7
GOS-3B	54.1	54.1	1.2	0.9	2.1	1.6
GOS-4	26.0	26.0	2.0	1.8	1.8	1.5
GOS-5	66.1	66.6	0.6	0.7	0.7	2.6
GOS-6	43.1	43.1	0.7	0.6	1.4	1.1

**Table 4:** Determination of TDF in GOS ingredients and precision data using both robust and classical statistics.

Sample	TDF concentration (g/100 g)		RSD (*r*) (%)		RSD (iR) (%)	
	Median	Mean	Robust	Classical	Robust	Classical
GOS-1A	22.2	21.2	0.5	1.0	1.1	2.2
GOS-1B	22.0	21.9	0.3	1.0	3.0	2.1
GOS-2	31.1	31.3	0.9	1.0	2.9	2.7
GOS-3A	31.7	31.7	1.1	1.1	2.7	1.8
GOS-3B	40.5	40.5	1.1	1.0	2.2	1.6
GOS-4	17.5	17.5	2.3	1.9	1.8	1.5
GOS-5	56.2	56.6	0.8	0.7	1.5	2.5
GOS-6	26.0	26.0	0.7	0.5	1.5	1.2

**Table 5:** Precision of GOS analysis in infant formula.

Sample	GOS concentration (g/100 g)		RSD (r) (%)		RSD (iR) (%)	
	Median	Mean	Robust	Classical	Robust	Classical
Lactogen 1^a^	2.70	2.69	0.8	0.9	1.1	1.2
Lactogen 2^a^	2.39	2.39	0.4	0.6	2.0	1.9
Lactogen 2^b^	2.58	2.58	2.4	2.0	3.5	2.7

^a^Samples analysed by HPLC-FLD method, *n* = 2 × 8. ^b^Samples analysed by HPAEC-PAD profiling method *n* = 2 × 9.

**Table 6:** Calculation of measurement uncertainty.

Sample	GOS content (g/100 g)^a^	RSD (iR) (%)	Recovery (%)	RSD_(Rec)_* (%)^b^	*u* (%)^c^	*U* (%)^d^
GOS-1A	43.5	2.7	117^f^	7.3	7.8	16
GOS-1B	43.7	2.7	120^f^	9.4	9.8	20
GOS-2	39.4	3.0	100	1.6	3.4	6.8
GOS-3A	42.4	2.5	110^f^	4.8	5.4	11
GOS-3B	54.1	2.1	105^f^	2.7	3.4	6.8
GOS-4	26.0	1.8	90.7^f^	6.4	6.7	13
GOS-5	66.1	0.7	84.2^f^	9.9	9.9	20
GOS-6	43.1	1.4	96.4	1.8	2.3	4.6
Spiked formula	2.63	2.0	102^f^	0.69	2.1	4.2
Lactogen 2	2.39	2.0	92.3^f^	4.9	5.3	11
Lactogen 2^e^	2.58	3.5	97.7	1.2	3.7	7.4

^a^Median GOS content as measured.  ^b^RSD_(Rec)_*: the relative standard deviation of the recovery, corrected if the recovery is not 100% and calculated as follows:

${{\text{SD}}_{\left(\text{Rec}\right)}}^{*}=\sqrt{{\left((1-\text{recovery})/2\right)}^{2}+{{\text{SD}}_{\left(\text{Rec}\right)}}^{2}}$ then, RSD_(Rec)_* = SD_(Rec )_*/(recovery) × 100.

^c^ *u*: relative measurement uncertainty expressed in % and calculated as follows:

$u=\sqrt{{\left(\text{RSD}(\text{iR})\right)}^{2}+{\left({{\text{RSD}}_{(\text{Rec)}}}^{*}\right)}^{2}}$.

^d^ *U*: expanded relative measurement uncertainty expressed as % and calculated as *U* = 2 × *u*.

^e^Results for this sample obtained using the HPAEC-PAD profiling method. ^f^Recovery is significantly different from 100%.

**Table 7:** Composition of different GOS ingredients in terms of oligosaccharide chain length.

Sample	Study	DP2^a^	DP3	DP4	DP5	DP6	DP7	DP8
GOS-1A	This study	48.9	39.1	6.19	2.20	1.64	1.39	0.641
GOS-1B	This study	49.1	38.9	6.18	2.19	1.66	1.37	0.610
GOS-2	This study	21.0	68.5	10.2	0.293			
GOS-3A	This study	25.1	63.1	11.4	0.403			
GOS-3B	This study	25.1	63.1	11.4	0.389			
GOS-4	This study	33.0	44.2	17.3	5.51			
GOS-5	This study	15.1	51.0	25.0	5.59	2.84	0.474	
GOS-6	This study	40.2	35.8	16.3	5.91	1.71	0.105	
GOS-H1^e,f^	Hernandez^b^	41.4*	37.0	14.4	7.23			
GOS-H2^e^	Hernandez^b^	61.6	36.0	2.4	0.00			
GOS-H3^e^	Hernandez^b^	36.8*	39.1	15.8	8.31			
GOS-C1^f^	Coulier^c^	40.4	32.5	15.9	7.2	2.8	0.9	0.3
GOS-A1^f^	Albrecht^d^	33.3	40.4	17.5	7.0	1.8		

DP: degree of polymerization; ^a^DP2 excludes lactose; ^b^data adapted from Hernández-Hernández et al. [11];  ^c^data adapted from Coulier et al. [10]; ^d^data adapted from Albrecht et al. [30]; ^e^GOS-H1, GOS-H2, and GOS-H3 are GOS-1, GOS-2, and GOS-3, respectively, in the original publication; ^f^GOS-H1, GOS-C1, and GOS-A1 are Vivinal GOS; *DP2 may be slightly underestimated since ß-D-Gal-(1→2)-D-Glc coelutes with lactose in this study.
